# Characterization of a *Pasteurella multocida* type A strain associated with a severe bronchopneumonia outbreak in gilts

**DOI:** 10.1186/s40813-026-00510-8

**Published:** 2026-04-11

**Authors:** Sophie Kähl, Helene Fenzl, Martin Dembowski, Regina Kröger, Claudia Hartig, Carla Heddier, Reiner Ulrich, Johannes Kauffold, Christoph Georg Baums

**Affiliations:** 1 Institute of Bacteriology and Mycology, Centre for Infectious Diseases, Faculty of Veterinary Medicine, Leipzig University, 04103 Leipzig, Germany; 2 Clinic for Ruminants and Swine, Faculty of Veterinary Medicine, Leipzig University, 04103 Leipzig, Germany; 3 Institute of Veterinary Pathology, Faculty of Veterinary Medicine, Leipzig University, 04103 Leipzig, Germany; 4 FGS Tierarztpraxis GmbH & Co.KG, 33142 Büren, Germany

**Keywords:** Bronchopneumonia, *Pasteurella multocida* serotype A, LPS, MLST, IgM, Pneumonic pasteurellosis, *Trueperella pyogenes*, Ide_*Ssuis*_

## Abstract

**Background:**

In pigs, *Pasteurella multocida* (*P. multocida*) serotypes A, B and D are causative agents of pneumonia, hemorrhagic septicemia and progressive rhinitis atrophicans, respectively. In the case of bronchopneumonia in pigs, *P. multocida* is generally regarded as an opportunistic pathogen following predisposing infectious agents. This study was initiated by an outbreak of bronchopneumonia in gilts associated with high mortality during lactation. Objectives of this study were to characterize the *P. multocida* isolate and systemic humoral immunity in gilts against this pathogen.

**Results:**

Bacteriological analyses revealed a high load of *P. multocida* and *Trueperella pyogenes* (*T. pyogenes*) in the lungs of diseased gilts, whereas other infectious agents such as viruses or *Mycoplasma* species were not found. The *P. multocida* isolate belonged to serotype A, sequence type 3, lipopolysaccharide genotype L3 and carried virulence-associated genes such as ompA, hgbA, tonB, ptfA, nanB and nanH. The lung isolate proliferated in porcine blood drawn from piglets and gilts of a non-affected farm, but showed reduced survival in blood drawn from gilts of the affected breeding farm, whereas the strain proliferated in blood of 6- and 8-week-old piglets. Significantly higher levels of IgG binding to the *P. multocida* isolate were detected in gilts of the affected farm in comparison to gilts of the unaffected herd. Gilts of both farms showed elevated levels of serum IgM binding to the *P. multocida* isolate in comparison to weaning piglets. Specific degradation of IgM in serum of gilts resulted in increased survival of this strain in reconstituted blood.

**Conclusion:**

The *P. multocida* serotype A isolate described in this manuscript is associated with severe bronchopneumonia in gilts as part of a mixed infection with *Trueperella pyogenes*, but without detection of predisposing viral or mycoplasmal infections. Our data suggests that affected gilts undergo a systemic immune response leading to increased specific IgG levels and bactericidal immunity. Furthermore, our in vitro results indicate that IgM plays an important role in mediating killing of this *P. multocida* strain in blood of gilts.

**Supplementary Information:**

The online version contains supplementary material available at 10.1186/s40813-026-00510-8.

## Background

*Pasteurella multocida* (*P. multocida*) is a Gram negative coccobacillus described as a commensal in most domestic and wild animals [1], but also as causative agent of severe pneumonia in pigs [2]. There are five capsular types which are associated with different diseases. Serotypes A and D are detected in cases of pneumonic pasteurellosis which is thought to follow primary infections with viral or other bacterial predisposing pathogens [3–5]. Toxinogenic strains of serotype D cause progressive atrophic rhinitis (PAR) and serotype B is mostly responsible for sporadic outbreaks of hemorrhagic septicemia (HS) [3, 6]. Pneumonia and PAR are most common in piglets and finisher swine, while HS affects all ages [3, 7]. Since 2010, hemorrhagic septicemia reemerged in Germany. *P. multocida* capsule type B was isolated from pigs, cattle, wild boars, fallow deer, red deer and a horse from 2010 to 2019 [8].

Experimental infection with *P. multocida* alone rarely leads to clinical signs or pathological lesions of pneumonia in pigs. Pretreatment with infectious or chemical predisposing factors are generally necessary to induce pneumonia [9]. This is in accordance with the opportunistic nature of *P. multocida* in the pathogenesis of pneumonia [3]. To date, no specific profile of virulence-associated factors other than biosynthesis genes of the serotype A capsule were associated with pneumonia. Both toxigenic and nontoxigenic strains are detected in acute cases [3]. PCRs for profiling of virulence-associated factors in *P. multocida* such as genes involved in siderophore uptake (*tonB*, *hgbA*) and adhesion (*ptfA*, *pfhA*) as well as toxins (*toxA*), sialidases (*nanB*, *nanH*) and outer membrane proteins (*ompA*, *ompH*,* oma87*,* plpB*) are described [10–14]. Siderophores and sialidases are important for iron acquisition and nutrition, respectively [15]. The mentioned adhesins are crucial for adherence, colonization and thus for the initial stages of mucosal infection [16].

In this study, a *P. multocida* isolate from an outbreak of severe pneumonia in gilts was genotypically and phenotypically characterized, in order to identify distinct features of a putative *P. multocida* pathotype causing pneumonia in gilts. Furthermore, IgG and IgM binding to *P. multocida* and bacterial survival in porcine blood was investigated to better understand its role in control of bacteremia and to figure out, if gilts in the affected herd go through a systemic immune response against this *P. multocida* strain.

## Methods

### Pathological and pathohistological investigations

In a farrow-to-finish farm with 3200 dams in East Germany (Saxony), farrowing gilts showed severe respiratory disease as described in the Results section. Two diseased gilts were euthanized for post-mortem examination. Following necropsy, samples from various organs including the brain, heart, lungs, spleen, liver and kidneys were collected for further histological and molecular investigations. The tissue samples were fixed in 4% neutral-buffered formaldehyde for at least 24 h, followed by embedding in paraffin wax. Sections of approximately 2 μm thickness were stained with hematoxylin and eosin as well as Taylor’s modified Gram-stain and examined using an Olympus BX53 microscope, equipped with WHN10X-H/22 eyepieces and 4x/0.13, 10x/0.30, 20x/0.50, 40x/0.75, and 100x/1.30oil objectives (Evident Europe GmbH, Hamburg, Germany). Digital images were captured using an Olympus DP28 color camera and processed with CellSens Dimension software version 4.3.

### Vaccination of gilts with an autogenous *Pasteurella multocida* bacterin

Starting in January 2024, gilts were prime-boost vaccinated six and three weeks pre farrowing with an autogenous *Pasteurella multocida* bacterin using the strain 23434 described in this study. The autogenous vaccine was generated by Vaxxinova Diagnostics GmbH.

### Molecular and bacteriological diagnostics

Lungs, spleen, kidneys, inguinal lymph nodes, mandibular lymph nodes and brain were examined molecular biologically for Classical swine fever (CSF) by RT-PCR and for African swine fever (ASF) and Aujeszky`s disease by PCR by the Saxony’s Health and Veterinary Research Institute (LUA, Leipzig, Germany). Additionally, lungs were examined by real-time PCR for porcine circovirus type 2, porcine cytomegalovirus, swine influenza virus, porcine reproductive and respiratory syndrome virus, porcine respiratory coronavirus, *Mycoplasma hyopneumoniae* (*M. hyopneumoniae*) and *Mycoplasma hyorhinis* by Vaxxinova Diagnostics GmbH (Leipzig, Germany). For bacteriology, samples of affected lung tissue were streaked out on Colombia blood agar plates and chocolate agar. After aerobic or anaerobic incubation for 48 h at 37 °C ± 1 °C, colonies were identified by MALDI TOF-MS.

### Molecular characterization

To characterize the *P. multocida* strain 23434, isolated from the lung of one of the examined gilts, the genes *cap*A and *cap*B encoding biosynthesis of capsules of type A and B strains, respectively, and further virulence-associated factors were differentiated by PCRs for *tonB*, *hgbA*, *ptfA*, *pfhA*, *toxA*, *nanB*, *nanH*,* ompA*, *ompH*,* oma87* and *plpB* [10, 17]. Strains 20,275 (from a wound of a cat) and 21,452 (from hemorrhagic septicemia in a pig [6]) were included for comparison. Genomic DNA was extracted by the Blood and Tissue Kit (Qiagen, Hilden, Germany) according to the manufacturers instruction and PCRs were conducted with OneTaq Quick-Load DNA Polymerase (NEB, Frankfurt am Main, Germany) as described [10, 17]. Genotyping was performed by multi-locus-sequence-typing (MLST) and lipopolysaccharide (LPS) outer core biosynthesis locus amplification. For MLST, PCRs on *P. multocida* strains 20275, 21452 and 23434 were conducted as described before using the Mulit-host MLST scheme and OneTaq Quick-Load DNA Polymerase or Q5^®^ High-Fidelity DNA Polymerase (NEB, Frankfurt am Main, Germany) according the manufacturer`s instruction [18]. After purification of amplification products with NucleoSpin Gel and PCR Clean-up Mini kit (Macherey-Nagel, Dueren, Germany), Sanger sequencing was conducted by Microsynth AG (Goettingen, Germany). Sequence types were determined by analyzing the resulting sequences with the MLST database website [19]. LPS typing of *P. multocida* strains 20275, 21452 and 23434 was performed by PCRs detecting eight different LPS genotypes as described before [20]. Briefly, using Q5^®^ High-Fidelity DNA Polymerase according to the manufacturer`s instructions (New England Biolabs, Frankfurt am Main, Germany), DNA was amplified with 30s initial denaturation followed by 30 cycles with 10s at 98 °C, 20s at 50 °C and 5 min at 72 °C and a final extension for 2 min at 72 °C.

### Bactericidal assay

The three *P. multocida* strains 23434, 20275 and 21452 (see above) were analyzed in an in vitro blood survival assay as described before [21]. Blood samples from five-, six- or eight-weeks old piglets (six piglets each) and seven- to nine-month-old gilts from the affected farm (farm A, six gilts) or an uninfected control farm (farm B, four gilts) were infected in vitro with 2.7-5.7 × 10^6^ CFU of the indicated *P. multocida* strains. Blood samples were drawn after the beginning of the outbreak but before starting of vaccination. Bacterial survival factors were calculated by dividing colony forming units (CFU) after two hours by CFU at inoculation time. Survival factors above 1 indicate survival or even proliferation of the inoculated bacteria, where bacteria were killed when survival factors were lower.

### Serological investigations

To investigate whether differences in killing between the farms were associated with differences in antibody levels, ELISA were conducted to measure IgM and IgG antibodies binding to the bacterial surface [21]. Briefly, 96-well-plates were coated with 0.5% formaldehyde-inactivated *P. multocida* 23434 bacteria overnight at 4 °C. Sera of three gilts of farm A were pooled and used as standard. Colostrum-deprived serum was used as negative control. Sera drawn from pigs of both farms investigated also in the blood survival assay were investigated in ELISA.

### IgM cleavage

Porcine sera were digested with recombinant IgM degrading enzyme of *Streptococcus suis* (*S. suis*), designated rIde_Ssuis_, a highly specific protease cleaving only porcine IgM [22]. Therefore, 250 µl serum was incubated at 37 °C for 2.5 h with 12.4 µg rIde_Ssuis_. As negative controls, sera were incubated with rIde_Ssuis__C195S, a non-functional point-mutated variant of Ide_Ssuis_ [23] or PBS. The digested sera were then used to reconstitute blood by adding blood cells of healthy pigs. Afterwards, bactericidal assays were conducted as described above with *P. multocida* 23434.

## Results

### Clinical background and pathology

This study was initiated by an outbreak of severe respiratory disease in farrowing gilts of a farrow-to-finish farm. Morbidity in gilts was estimated to be around 85% by the veterinarian managing this herd. Clinical signs were severe cough, dyspnea, fever (40.2–40.4 °C) for four to five days and lethargy. As shown in Table 1, a high mortality of 8.6% was observed in lactating gilts during the outbreak in 2023. This outbreak increased over a period of about three month and after vaccination with an autogenous bacterin including only the isolated *P. multocida* strain morbidity and mortality decreased. In the following two years, mortality in gilts decreased from 8.6% to 3.4%.

**Table 1:** Mortality of gilts in the affected farm A from 2023 to 2025

	2023	2024	2025
Deceased	5.8%	2.4%	2.0%
Killed	2.8%	2.2%	1.4%
Total	8.6%	4.6%	3.4%

**Fig. 1:**
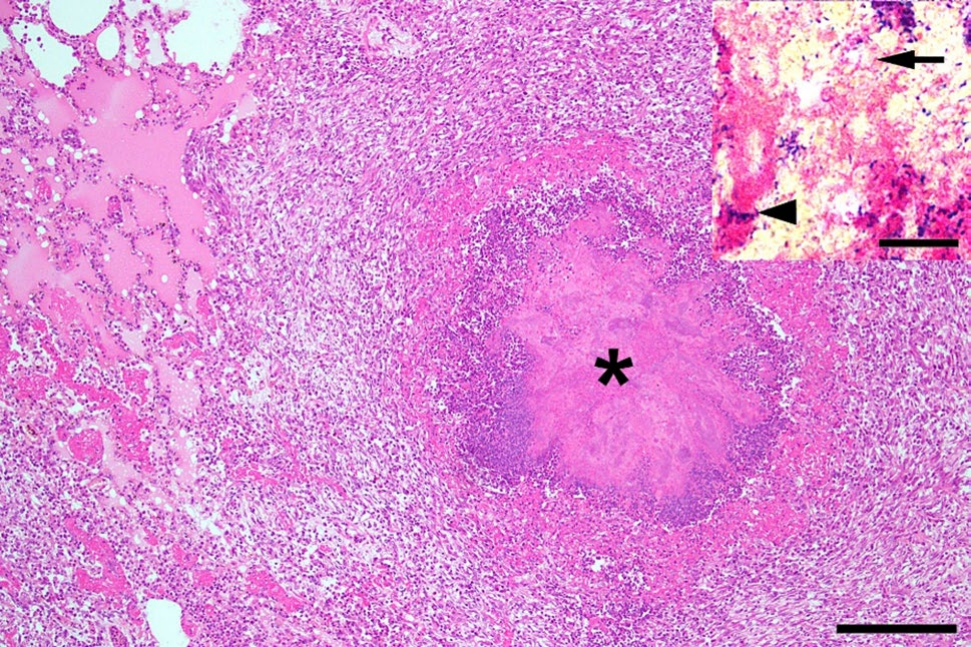
Chronic suppurative-to-pyogranulomatous bronchopneumonia with abscessation in a gilt humanely killed during the outbreak in 2023. Lung: severe, chronic, coalescing purulent bronchopneumonia with abscess formation (*). Hematoxylin-Eosin; Bar = 200 μm. Inset: High numbers of fine, coccobacillary, Gram-negative bacteria (arrow) and larger, rod-shaped, Gram-positive bacteria (arrowhead). Taylor’s modified Gram-stain; Bar = 10 μm

The lungs of two investigated, severely diseased gilts revealed slightly different histopathological findings. One gilt had moderate, acute-to-subacute, coalescing, purulent bronchopneumonia in the accessory lobe and mild, subacute, multifocal lymphohistiocytic interstitial pneumonia. The second gilt had a severe, chronic suppurative-to-pyogranulomatous bronchopneumonia with abscessation in the right caudal lobe (Fig. 1). The abscesses were demarcated by granulation tissue containing macrophages, lymphocytes, plasma cells, neutrophils, and a few foreign-body-type multinucleated giant cells surrounding a central mass of degenerated neutrophils and bacterial colonies. Further histopathological findings included type 2 pneumocyte hyperplasia, alveolar histiocytosis, and alveolar and interstitial edema with fibrin thrombi. There was also fibroblastic to fibrino-purulent pleuritis. Using Taylor’s modified Gram stain, the bacteria were characterized as Gram-negative coccobacilli and Gram-positive rods (inset in Fig. 1). Histopathological examination of other organs revealed a mild lymphohistioplasmacytic interstitial nephritis and a mild suppurative and lymphohistioplasmacytic chorioiditis in the second gilt, whereas the other one displayed no relevant changes in brain, heart, spleen, liver and kidneys.

### Molecular and microbiological investigations

CSFV, ASFV, SHV-1, porcine circovirus type 2, porcine cytomegalovirus, swine influenza virus, porcine reproductive and respiratory syndrome virus, porcine respiratory coronavirus, *M. hyopneumoniae* and *Mycoplasma hyorhinis* were not detected in the examined samples of the two necropsied gilts.

**Fig. 2:**
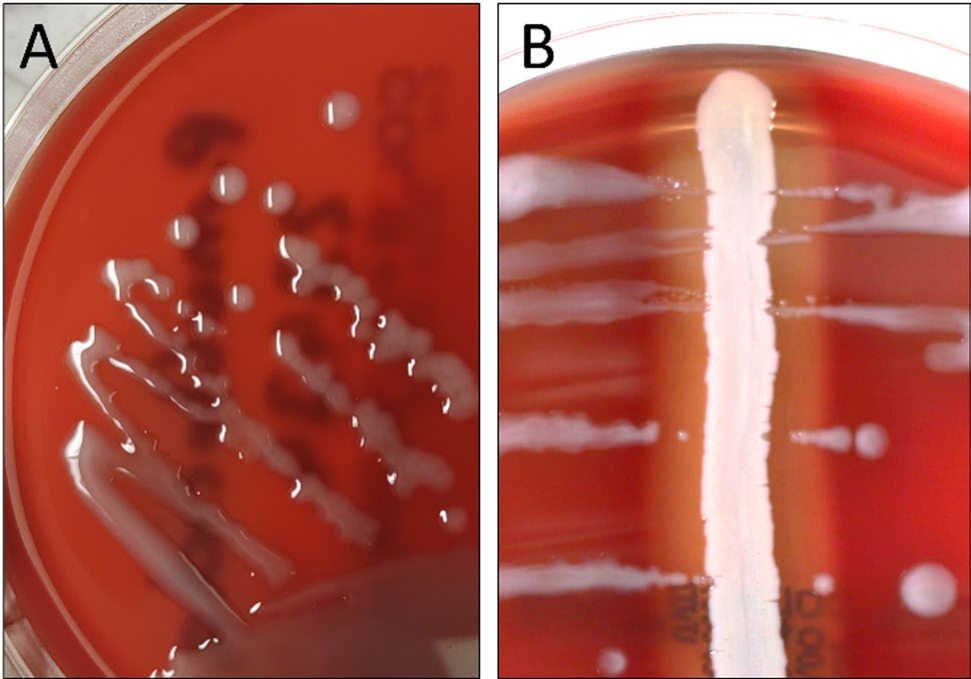
*P. multocida* 23434 isolated from lung tissue of a gilt with severe bronchopneumonia (**A**) Mucous phenotype. (**B**) Hyaluronidase test with *Staphylococcus aureus* revealed hyaluronic acid in capsule of *P. multocida* 23434

High numbers of *P. multocida* and *Trueperella pyogenes* (*T. pyogenes*) were detected culturally in the lungs of both gilts. *P. multocida* isolates showed an intense mucous phenotype and were positive in the hyaluronidase test with *Staphylococcus aureus* (Fig. 2) [24]. Further investigations were conducted with *P. multocida* 23434 isolated from the lungs of one of the two gilts.

### Molecular characterization

**Fig. 3:**
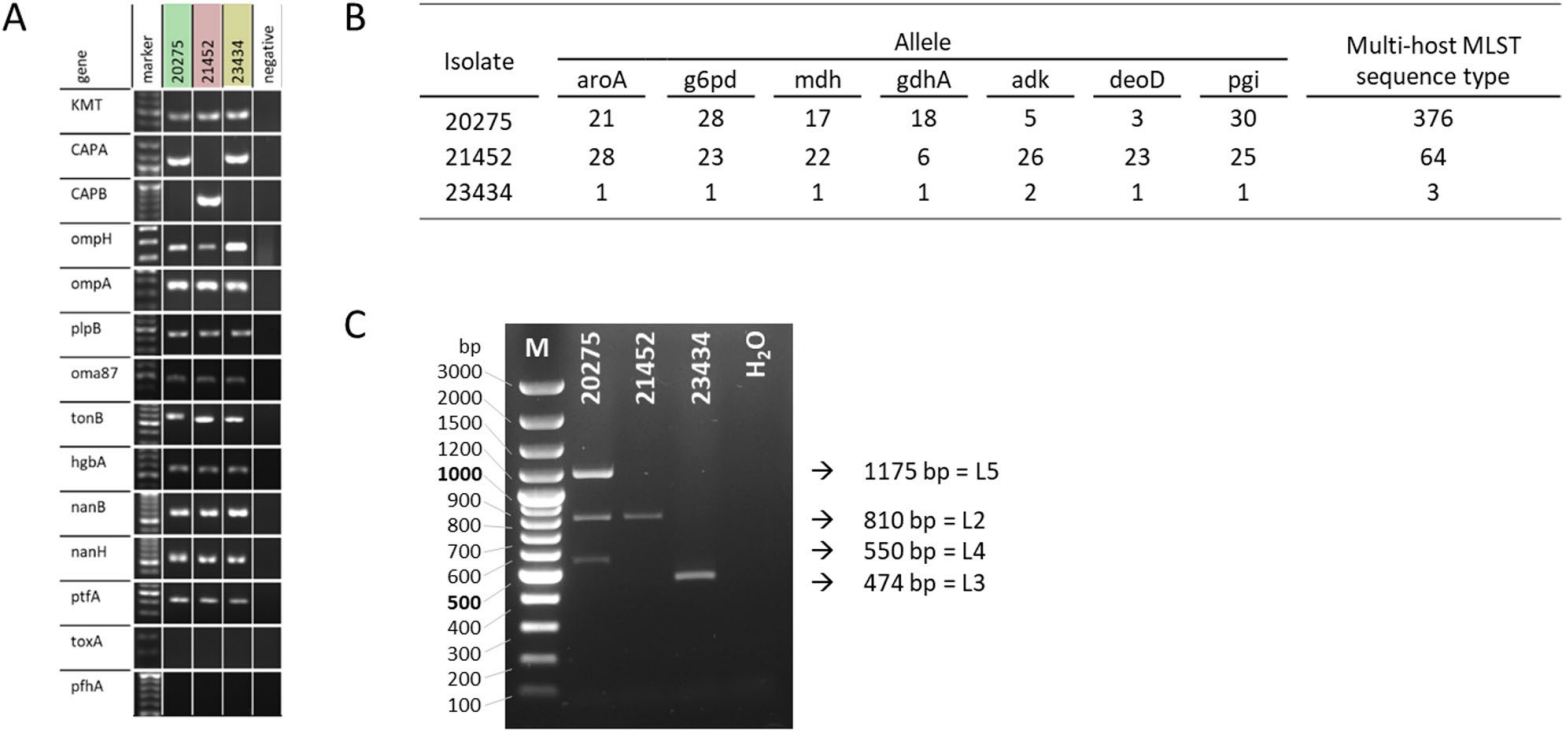
Genotyping of *P. multocida* isolated from a diseased gilt described in this study (23434), a feline wound isolate (20275) and a hemorrhagic septicemia strain (21452). (**A**) Strains 23434 and 20275 were confirmed as *P. multocida* (KMT) capsule type A (CAPA) and strain 21452 was found to be a capsule type B (CAPB). All investigated strains harbored several virulence-associated genes: *ompH*,* ompA*,* oma87*,* plpB* (outer membrane proteins); *hgbA*,* tonB* (iron acquisition); *ptfA* (fimbriae, adhesin); *nanB*,* nanH* (neuraminidases). *pfhA* (hemolysin, adhesin) and *toxA* (dermonecrotic toxin, atrophic rhinitis) could not be detected. (**B**) Results of MLST analysis of the indicated *P. multocida* strains. **C**). Differentiation of LPS genotypes of *P. multocida* isolates by PCR

The *P. multocida* lung isolate of a diseased gilt (23434) was compared with a feline wound isolate (20275) and a strain isolated from a pig with hemorrhagic septicemia (21452 [6]), . All three strains were confirmed as *P. multocida* (KMT) and strain 23434 as well as strain 20275 were assigned to capsular type A (CAPA). Except of *toxA* (*P. multocida* toxin involved in PAR) and *pfhA* (hemagglutinin, adhesin), all investigated genes were detected in the three strains, including genes encoding outer membrane proteins (*ompH*,* ompA*,* oma87*,* plpB*), iron acquisition related proteins (*hgbA*,* tonB*), fimbriae (*ptfA*) and neuraminidases (*nanB*,* nanH*). Thus, there was no difference between these three strains in the profile of the investigated virulence-associated genes except for the capsule biosynthesis gene CAPA (Fig. 3A).

MLST revealed, that *P. multocida* 23434 and 21452 belong to ST3 and ST64, respectively (Fig. 3B). As strain 20275 could not be assigned to any existing sequence type, the new ST376 has been submitted to the MLST database (Fig. 3B). Furthermore, LPS genotypes were determined by PCR, where amplicons of the predicted size were detected for L2 and L3 using genomic DNA of strain 21452 and 23434, respectively. Strain 20275 could not be assigned to one LPS genotype, as PCR revealed amplicons corresponding to L5, L2 and L3 (Fig. 3C).

### Bacterial survival in porcine blood

**Fig. 4:**
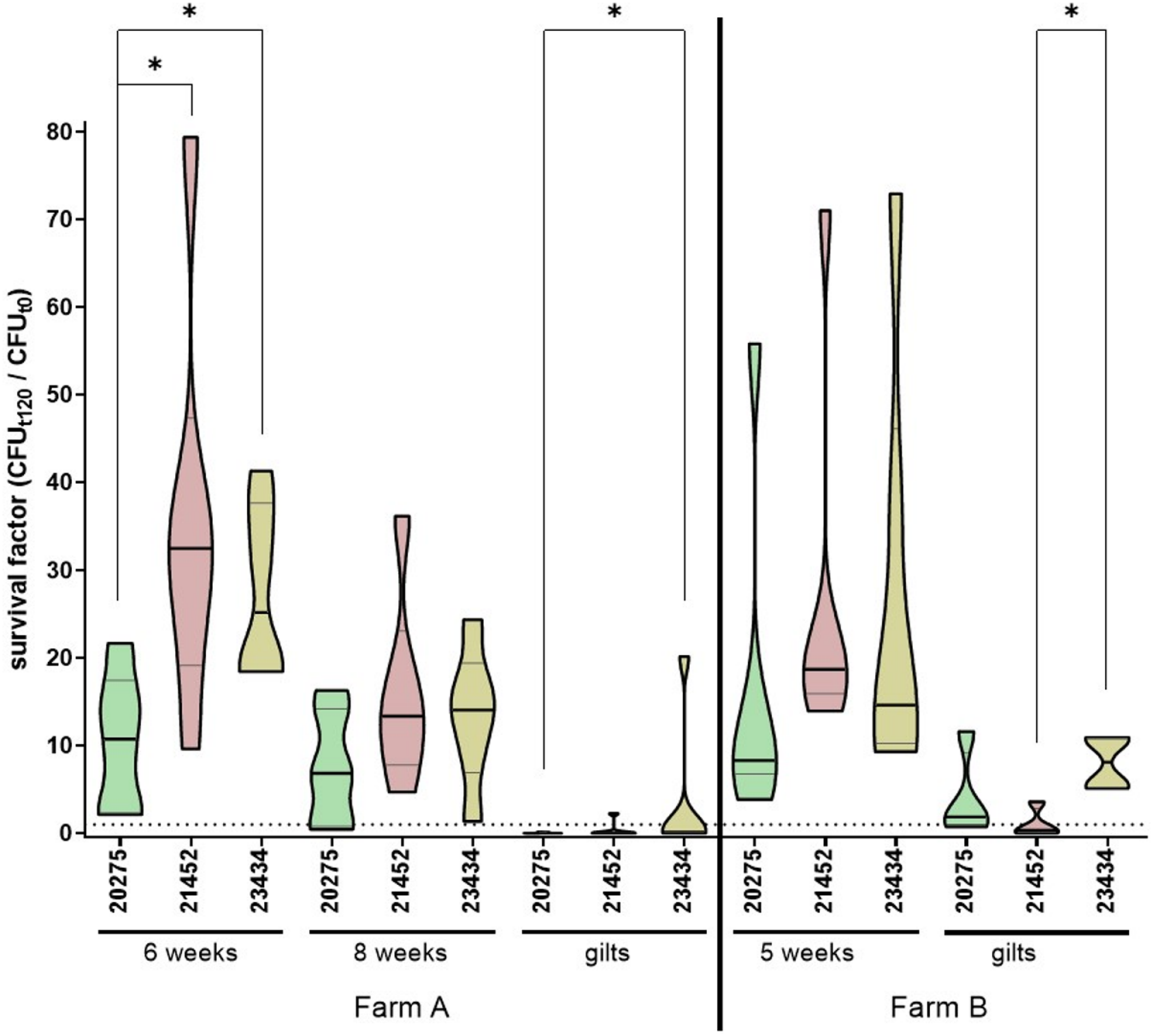
*P. multocida* 23434 was killed in blood from most gilts from the outbreak Farm **A**, but proliferated in whole blood drawn from all gilts and piglets from Farm **B** investigated for comparison. Whole blood drawn from piglets and gilts from the affected farm A and the control farm B was inoculated with the *P. multocida* 23434 isolated from farm A. For comparison, *P. multocida* 20275 (wound of a cat) and 21452 (hemorrhagic septicemia [6]) were also investigated. Median (black line) and quartiles (grey lines) are indicated. A survival factor of 1 (dotted line) indicates that the number of bacteria did not change during the two hours of incubation. Values above and below the dotted lines indicate proliferation and killing of *P. multocida*, respectively. Statistical analysis was done by ANOVA and Dunn’s multiple comparisons test (*: *p* ≤ 0.05)

In the affected farm A as well as for comparison in a non-affected farm B, we collected blood from piglets and gilts after the described outbreak but before introduction of the autogenous *P. multocida* vaccination. This blood was infected in vitro with the *P. multocida* outbreak strain 23434 and the two other *P. multocida* strains to assess bactericidal immunity against *P. multocida* in different units of this farm. As shown in Fig. 4, survival factors of all three strains were higher in piglets than in gilts. Strain 23434 was killed in blood from four of six gilts of the affected farm A, whereas this isolate proliferated in all tested gilts of the control farm B. Furthermore, *P. multocida* 23434 was the strain with the highest median level of proliferation in blood of gilts of farms A and B. Regarding the non-infected farm B, differences between *P. multocida* 23434 and 21452 were significant (Fig. 4). This indicates a more efficient immune evasion of strain 23434 in blood of presumably non-infected gilts in comparison to the *P. multocida* strain 21452, which was originally isolated from a case of hemorrhagic septicemia.

### IgG and IgM in porcine serum

**Fig. 5:**
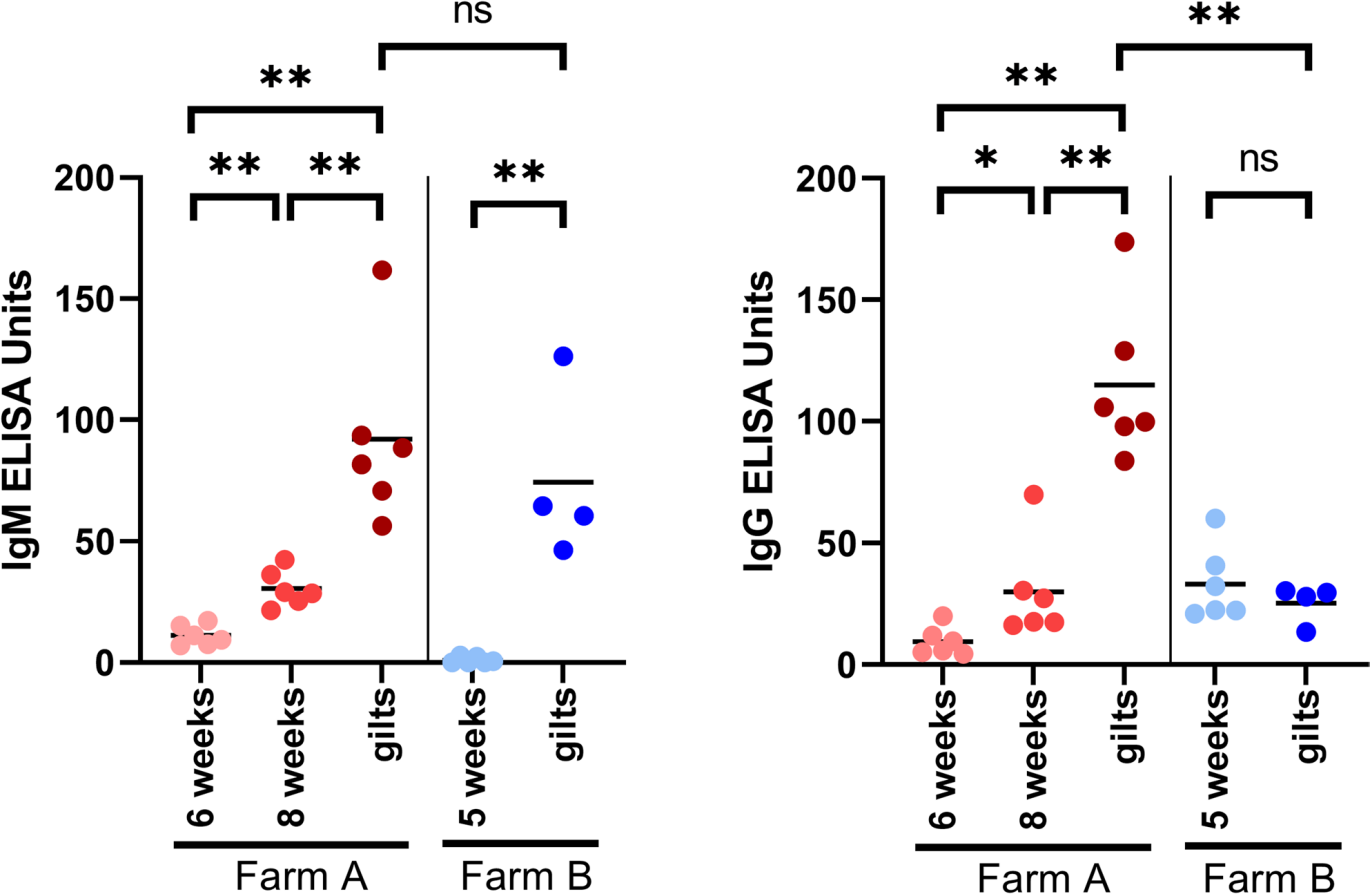
Levels of IgM and IgG binding to *P. multocida* 23434 in pigs of the indicated age of farm A (history of bronchopneumonia in gilts) and farm B. Levels of immunoglobulins were measured by ELISA with immobilized, inactivated bacteria of strain 23434 as antigen. Statistical analysis was done with Mann-Whitney test (ns: *p* ≥ 0.05; *: *p* < 0.05; **: *p* < 0.01; ***: *p* < 0.001)

Levels of IgM binding to *P. multocida* 23434 were comparable between farm A and farm B (Fig. 5). However, the gilts from the affected farm A showed significantly higher specific IgG levels than the gilts from farm B, which is in accordance with the low bacterial survival factors in respective blood samples.

### IgM-depended killing in porcine blood

**Fig. 6:**
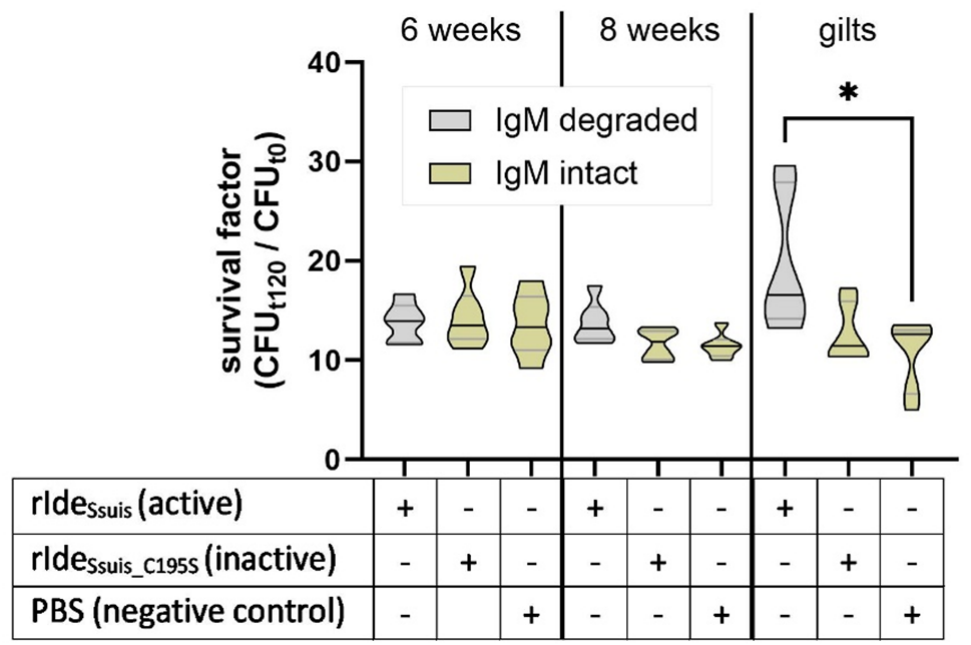
Killing of *P. multocida* 23434 is IgM-mediated in blood drawn from gilts. IgM was degraded in serum drawn from piglets and gilts of outbreak farm A through incubation with rIde_Ssuis_. As negative controls, sera were incubated with non-functional rIde_Ssuis__C195S (point mutated rIde_Ssuis_) or PBS. Blood was reconstituted with pretreated sera and freshly collected porcine blood cells and infected with *P. multocida* 23434 in vitro. Bacterial survival factors were calculated by dividing CFU after two hours incubation at 37 °C by CFU at inoculation time. *P. multocida* was able to proliferate significantly more in blood with serum from gilts pretreated with rIde_Ssuis_. In piglets, no significant differences were observed between the different treatments. Statistical analysis was done with Mann-Whitney test (*: *p* < 0.05)

To access the role of IgM in control of bacteremia, we specifically degraded IgM in the serum samples by addition of the highly specific IgM protease Ide_*Ssuis*_. In gilts the digestion leads to a significantly increased bacterial survival (Fig. 6), indicating that the high IgM levels in gilts (Fig. 5) play a role in controlling bacteremia. IgM cleavage did not result in significant differences in survival of *P. multocida* 23434 in blood of six- or eight-weeks old piglets.

## Discussion

*P. multocida* is known as a causative agent of numerous diseases in several species, including pneumonia, atrophic rhinitis, meningitis, fowl cholera, endocarditis, wound infections and hemorrhagic septicemia [1, 3]. In spite of several attempts to develop a pneumonia model, experimental application of *P. multocida* alone often failed to lead to clinical signs or pathologies of pneumonia in pigs and pretreatment with endotoxin, mycotoxin, ammonia or coinfection with *M. hyopneumoniae*, PRRSV, *Bordetella bronchiseptica* or other predisposing or primary pathogens were necessary to induce pneumonia [9]. However, Oliveira Filho et al. 2015 reported successful experimental intranasal infection of pigs with a porcine *P. multocida* type A strain isolated from a finishing pig with severe respiratory disease and fibrinosuppurative pleuropneumonia leading to fibrinous pleuritis, suppurative bronchopneumonia, pleuropneumonia and septicemias. These animals were tested negative for other main respiratory pathogens [25]. Furthermore, the same authors observed hyperthermia, dyspnea, cough and several macroscopic and microscopic lesions in lungs after experimental infection of pigs with eight different *P. multocida* type A strains [26]. Their results suggested that the pfhA region might be related to pathogenicity. However, pfhA was not detected in the *P. multocida* strain 23434 investigated in this study, which indicates that the pfhA locus is not essential for causing severe respiratory infections in gilts. The bronchopneumonia in gilts described here was also associated with a type A strain. As both molecular biological analyses of the examinated gilts and serological investigations of the affected farm in the months following the outbreak regarding antibodies to PCV2, PRRSV, APP and *M. hyopneumoniae* revealed negative results, predisposition by those pathogens is unlikely. Nevertheless, environmental factors such as draught, temperature, humidity and ammonia were not examined, which is why a predisposition by those impacts could not be rule out.

Here, a severe *P. multocida* outbreak was observed in gilts showing high fever, cough and dyspnea. Bacteriological and molecular investigations revealed no other respiratory infectious agents other than *P. multocida* and *T. pyogenes*. *T. pyogenes* is part of the upper respiratory microbiota and an opportunistic pathogen [27]. Ascending infection might increase inflammation of the bronchiolar epithelium and lead to suppurative lesions [3]. Bronchopneumonia associated with *T. pyogenes* is described as febrile and fatal disease [3]. The observed pathological morphology is in accordance with abscessing lesions caused by *T. pyogenes* [28]. Nevertheless, this bacterium is not yet described as a primary pathogen for lung diseases on a herd level. Instead, the mainly described diseases are metritis, endometritis and mastitis in cattle and mild cough, pneumonia and elevated body temperature in pigs [28–30]. Purulent and abscessing infections of the lung are described in different animals, but not within the context of outbreaks, which would be defined as infection of several individuals with the same bacterial strain [28]. Wente et al. investigated sixteen dairy herds with several *T. pyogenes* isolates from mastitis and found that almost no or only two cows were infected with the same strain. This indicates, that the contagiousness of *T. pyogenes* appears to be quite low and an outbreak on a herd level is unlikely [31]. Furthermore, after vaccination with an autogenous bacterin including only the isolated *P. multocida* strain clinical signs improved and mortality decreased. Accordingly, we speculate that *T. pyogenes* caused a secondary infection that might have been triggered by the tissue damage caused by *P. multocida*. Nevertheless, the contribution of *T. pyogenes* to this case could not be certainly determined based on our data.

PCR profiling of virulence genes revealed no genotypic differences between the three investigated *P. multocida* strains originating from different diseases. For *ptfA*, *ompA*, *ompH*, *plpB*, *tonB* and *hgbA* this is in accordance with results of Prajapati et al., who found these genes also in the genomes of all 30 investigated porcine type A *P. multocida* strains. The genes *nanB*, *nanH* and *oma87* were found in 97% and *toxA* was not detected in any strain [32]. This suggests, that the described gene profile is not distinct from the profile of other *P. multocida* strains associated with other diseases. Of note, expression of Oma87, TonB, and NanH is significantly more upregulated in a highly virulent strain in comparison to a low virulent strain in lung tissue [33]. However, most of these genes are highly prevalent in *P. multocida* of porcine origin [4, 32]. In cattle, *pfhA*, *hgbB* and *tpbA* are significantly associated with disease. However, in pigs, only a *toxA* positive genotype indicates virulence with regard to PAR [4]. Overall, the significance of profiling virulence-associated genes of *P. multocida* in porcine practice is limited. We are not aware of a MP-PCR that allows to differentiate respiratory isolates of *P. multocida* in accordance with their virulence, except for toxinogenic strains associated with PAR.

Further genotyping assigned *P. multocida* 23434 to ST3, using the Multi-host MLST scheme. Based on the *P. multocida* MLST database, this sequence type was repeatedly isolated from pigs and cattle in association with pneumonia in different countries such as the United Kingdom, Canada, Spain, South Korea, China and Taiwan [34]. In this database, 143 porcine isolates associated with pneumonia were deposited, including 35 (24.5%) ST3 isolates. *P. multocida* 21275, which was isolated from an outbreak of hemorrhagic septicemia, was assigned to capsular type B and ST64. This is in accordance with the MLST database, as 62.4% of the deposited isolates from hemorrhagic septicemia belong to this sequence type, and previous studies [8, 34]. LPS genotyping revealed type L3 for the *P. multocida* strain 23434, which is in line with a previous study finding the A: L3 genotype in 25.00%, D: L6 in 42.86% and A: L6 in 23.21% of porcine isolates. In samples of pigs with respiratory disorders in China and Taiwan, A:L3 was found in 24.05% and 19.5% of cases, respectively [35]. The suggested increased virulence of the combined MLST: LPS genotype ST3:L3 found in strain 23434 is furthermore in accordance with a previous study, as Peng et al. analyzed 40 isolates of porcine pneumonia and found seven ST3 stains, all belonging to LPS genotype L3. Nevertheless, the majority of the investigated strains were assigned to ST10:L6 and ST11:L6 with 13 and 18 isolates, respectively, indicating a slightly higher significance for respiratory diseases in pigs so far [36].

We accessed survival of *P. multocida* strain 23434 in blood drawn from piglets of different ages. Generally, many pathogenic bacteria are efficiently killed in vitro in blood of pigs older than 10 weeks, as shown for *Klebsiella pneumoniae* [37], and *S. suis* [38]. An important finding of this study is the proliferation of *P. multocida* strain 23434 in blood of gilts previously not exposed to this pathogen (Fig. 4), highlighting the potential relevance of *P. multocida* for severe infections. This was a distinct characteristic, e.g. in comparison to strain 21452, originally isolated from a case of hemorrhagic septicemia [6].

On the other hand, the hemorrhagic septicemia strain proliferated in blood of younger piglets very efficiently. This suggests age-related differences in susceptibility to different *P. multocida* strains. In the case farm, blood of four of six gilts were able to kill all investigated strains. In contrast, in the unaffected farm B, *P. multocida* 23434 proliferated in the blood of all four gilts. This may be due to lower specific IgG levels in gilts of farm B as the levels of IgM binding to *P. multocida* were comparable. Further results indicate that IgM plays an important role in gilts but not in 6- and 8-week-old piglets. This is in contrast to *S. suis* bacteremia which is mainly controlled in 6- to 8- week old piglets by IgM [21, 38].

## Conclusions

Our investigations suggest that the distinct *P. multocida* type A strain might cause severe bronchopneumonia as part of a mixed infection with *T. pyogenes* in farrowing and lactating gilts. This is in association with survival in blood drawn from gilts, as gilts with high IgG levels against *P. multocida* were able to kill the bacteria in their blood in vitro. Degradation of IgM leads to increased bacterial survival, indicating that IgM is crucial for killing of this pathotype in blood of gilts. Further studies are warranted to identify distinct genetic markers of this pathotype.

## Supplementary Information

Below is the link to the electronic supplementary material.

Supplementary Material 1

## Data Availability

The datasets used and/or analyzed during the current study are available from the corresponding author on reasonable request.

## References

[1] Wilson BA, Ho M. Pasteurella multocida: from Zoonosis to Cellular Microbiology. Clin Microbiol Rev. 2013;26:631–55. 23824375 10.1128/CMR.00024-13 PMC3719492

[2] Davies RL, MacCorquodale R, Baillie S, Caffrey B. Characterization and comparison of Pasteurella multocida strains associated with porcine pneumonia and atrophic rhinitis. J Med Microbiol. 2003;52:59–67. 10.1099/jmm.0.05019-0. 12488567 10.1099/jmm.0.05019-0

[3] Zimmerman JJ, Karriker LA, Ramirez A, Schwartz KJ, Stevenson GW, Zhang J. Diseases of swine. 11th ed. Hoboken, NJ: Wiley-Blackwell; 2019.

[4] Ewers C, Lübke-Becker A, Bethe A, Kiebling S, Filter M, Wieler LH. Virulence genotype of Pasteurella multocida strains isolated from different hosts with various disease status. Vet Microbiol. 2006;114:304–17. 10.1016/j.vetmic.2005.12.012. 16427218 10.1016/j.vetmic.2005.12.012

[5] Carvalho LF, Segalés J, Pijoan C. Effect of porcine reproductive and respiratory syndrome virus on subsequent Pasteurella multocida challenge in pigs. Vet Microbiol. 1997;55:241–6. 10.1016/s0378-1135(96)01324-7. 9220619 10.1016/S0378-1135(96)01324-7 PMC7117206

[6] Soike D, Schulze C, Kutzer P, Ewert B, van der Grinten E, Schliephake A, et al. Akute Pasteurellose bei Damwild, Rindern und Schweinen in einer Region im Osten Deutschlands. [Acute pasteurellosis in fallow deer, cattle and pigs in a region of Eastern Germany]. Berl Munch Tierarztl Wochenschr. 2012;125:122–8. 22515030

[7] Cameron RD, O’Boyle D, Frost AJ, Gordon AN, Fegan N. An outbreak of haemorrhagic septicaemia associated with Pasteurella multocida subsp gallicida in large pig herd. Aust Vet J. 1996;73:27–9. 10.1111/j.1751-0813.1996.tb09949.x. 8660187 10.1111/j.1751-0813.1996.tb09949.x

[8] Kutzer P, Szentiks CA, Bock S, Fritsch G, Magyar T, Schulze C, et al. Re-emergence and spread of haemorrhagic septicaemia in Germany: the wolf as a vector? Microorganisms. 2021. 10.3390/microorganisms9091999. 10.3390/microorganisms9091999 PMC8465458 34576894

[9] Ross RF. Pasteurella multocida and its role in porcine pneumonia. Anim Health Res Rev. 2006;7:13–29. 10.1017/S1466252307001211. 17389051 10.1017/S1466252307001211

[10] Rajkhowa S. Development of a novel multiplex PCR assay for rapid detection of virulence associated genes of Pasteurella multocida from pigs. Lett Appl Microbiol. 2015;61:293–8. 10.1111/lam.12453. 26095172 10.1111/lam.12453

[11] Devi LB, Bora DP, Das SK, Sharma RK, Mukherjee S, Hazarika RA. Virulence gene profiling of porcine Pasteurella multocida isolates of Assam. Vet World. 2018;11:348–54. 10.14202/vetworld.2018.348-354. 29657428 10.14202/vetworld.2018.348-354 PMC5891851

[12] Farahani MF, Esmaelizad M, Jabbari AR. Investigation of iron uptake and virulence gene factors (fur, tonB, exbD, exbB, hgbA, hgbB1, hgbB2 and tbpA) among isolates of Pasteurella multocida from Iran. Iran J Microbiol. 2019;11:191–7. 31523401 PMC6711875

[13] Katsuda K, Hoshinoo K, Ueno Y, Kohmoto M, Mikami O. Virulence genes and antimicrobial susceptibility in Pasteurella multocida isolates from calves. Vet Microbiol. 2013;167:737–41. 10.1016/j.vetmic.2013.09.029. 24139632 10.1016/j.vetmic.2013.09.029

[14] Pintér K, Domán M, Wehmann E, Makrai L, Gantelet H, Magyar T. Correlations between virulence gene profiles and other genetic factors of Pasteurella multocida strains isolated from various host species. Vet Microbiol. 2025;308:110657. 10.1016/j.vetmic.2025.110657. 40768995 10.1016/j.vetmic.2025.110657

[15] Corfield T. Bacterial sialidases - Roles in Pathogenicity and Nutrition. Glycobiology. 1992;2:509–21. 10.1093/glycob/2.6.509. 1472757 10.1093/glycob/2.6.509

[16] Ziagham A, Gharibi D, Mosallanejad B, Avizeh R. Molecular characterization of Pasteurella multocida from cats and antibiotic sensitivity of the isolates. Vet Med Sci. 2024;10:e1424. 10.1002/vms3.1424. 38519838 10.1002/vms3.1424 PMC10959823

[17] Townsend KM, Boyce JD, Chung JY, Frost AJ, Adler B. Genetic organization of Pasteurella multocida cap Loci and development of a multiplex capsular PCR typing system. J Clin Microbiol. 2001;39:924–9. 10.1128/JCM.39.3.924-929.2001. 11230405 10.1128/JCM.39.3.924-929.2001 PMC87851

[18] Jolley KA, Bray JE, Maiden MCJ. Open-access bacterial population genomics: BIGSdb software, the PubMLST.org website and their applications. Wellcome Open Res. 2018;3:124. 10.12688/wellcomeopenres.14826.1. 30345391 10.12688/wellcomeopenres.14826.1 PMC6192448

[19] Pasteurella multocida typing. 07.01.2026. https://pubmlst.org/bigsdb?db=pubmlst_pmultocida_seqdef. Accessed 7 Jan 2026.

[20] Harper M, John M, Turni C, Edmunds M, St Michael F, Adler B, et al. Development of a rapid multiplex PCR assay to genotype Pasteurella multocida strains by use of the lipopolysaccharide outer core biosynthesis locus. J Clin Microbiol. 2015;53:477–85. 10.1128/JCM.02824-14. 25428149 10.1128/JCM.02824-14 PMC4298526

[21] Mayer L, Bornemann N, Lehnert S, Greeff A, de, Strutzberg-Minder K, Rieckmann K, Baums CG. Survival patterns of Streptococcus suis serotypes 1 and 14 in porcine blood indicate cross-reactive bactericidal antibodies in naturally infected pigs. Vet Microbiol. 2021;260:109183. 10.1016/j.vetmic.2021.109183. 34304027 10.1016/j.vetmic.2021.109183

[22] Seele J, Singpiel A, Spoerry C, von Pawel-Rammingen U, Valentin-Weigand P, Baums CG. Identification of a novel host-specific IgM protease in Streptococcus suis. J Bacteriol. 2013;195:930–40. 10.1128/JB.01875-12. 23243300 10.1128/JB.01875-12 PMC3571317

[23] Rungelrath V, Weiße C, Schütze N, Müller U, Meurer M, Rohde M, et al. IgM cleavage by Streptococcus suis reduces IgM bound to the bacterial surface and is a novel complement evasion mechanism. Virulence. 2018;9:1314–37. 10.1080/21505594.2018.1496778. 30001174 10.1080/21505594.2018.1496778 PMC6177247

[24] Carter GR, Rundell SW. Identification of type A strains of P multocida using staphylococcal hyaluronidase. Vet Rec. 1975;96:343. 10.1136/vr.96.15.343. 1125106 10.1136/vr.96.15.343

[25] de Oliveira Filho JX, Morés MA, Rebelatto R, Agnol AM, Plieski CL, Klein CS, et al. Pasteurella multocida type A as the primary agent of pneumonia and septicaemia in pigs. Pesq Vet Bras. 2015;35:716–24. 10.1590/S0100-736X2015000800003.

[26] de Oliveira Filho JX, Morés MAZ, Rebellato R, Kich JD, Cantão ME, Klein CS, et al. Pathogenic variability among Pasteurella multocida type A isolates from Brazilian pig farms. BMC Vet Res. 2018;14:244. 10.1186/s12917-018-1565-2. 30134904 10.1186/s12917-018-1565-2 PMC6103967

[27] Ribeiro MG, Risseti RM, Bolaños CAD, Caffaro KA, de Morais ACB, Lara GHB, et al. Trueperella pyogenes multispecies infections in domestic animals: a retrospective study of 144 cases (2002 to 2012). Vet Q. 2015;35:82–7. 10.1080/01652176.2015.1022667. 25793626 10.1080/01652176.2015.1022667

[28] Rzewuska M, Kwiecień E, Chrobak-Chmiel D, Kizerwetter-Świda M, Stefańska I, Gieryńska M. Pathogenicity and Virulence of Trueperella pyogenes: A Review. Int J Mol Sci. 2019. 10.3390/ijms20112737. 31167367 10.3390/ijms20112737 PMC6600626

[29] Qin L, Meng F, He H, Yang Y-B, Wang G, Tang Y-D, et al. A Virulent Trueperella pyogenes Isolate, Which Causes Severe Bronchoconstriction in Porcine Precision-Cut Lung Slices. Front Vet Sci. 2021;8:824349. 10.3389/fvets.2021.824349. 35174243 10.3389/fvets.2021.824349 PMC8841747

[30] Dong W-L, Liu L, Odah KA, Atiah LA, Gao Y-H, Kong L-C, Ma H-X. Antimicrobial resistance and presence of virulence factor genes in Trueperella pyogenes isolated from pig lungs with pneumonia. Trop Anim Health Prod. 2019;51:2099–103. 10.1007/s11250-019-01916-z. 31104226 10.1007/s11250-019-01916-z

[31] Wente N, Leimbach S, Woudstra S, Krömker V. Trueperella Pyogenes-Strain Diversity and Occurrence in Dairy Herds. Pathogens. 2024. 10.3390/pathogens13070534. 39057761 10.3390/pathogens13070534 PMC11279676

[32] Prajapati A, Yogisharadhya R, Mohanty NN, Mendem SK, Chanda MM, Siddaramappa S, Shivachandra SB. Comparative genome analysis of Pasteurella multocida strains of porcine origin. Genome. 2024;67:13–23. 10.1139/gen-2023-0021. 37639729 10.1139/gen-2023-0021

[33] Cheng Y, Wang K, Lin L, Zhao X, Pan Z, Zhou Z. Differences in pathogenicity and virulence-associated gene expression among Pasteurella multocida strains with high and low virulence in a lung tissue model. Microb Pathog. 2020;140:103911. 10.1016/j.micpath.2019.103911. 31830580 10.1016/j.micpath.2019.103911

[34] MLST database. 07.01.2026. https://pubmlst.org/bigsdb?db=pubmlst_pmultocida_isolates%26page=query%26prov_field1=f_country%26prov_value1=Germany%26submit=1. Accessed 7 Jan 2026.

[35] Wu C-F, Liao C-C, Chou C-C, Wang C-M, Huang S-W, Kuo H-C. Serovar and multilocus sequence typing analysis of Pasteurella multocida from diseased pigs in Taiwan. BMC Vet Res. 2025;21:117. 10.1186/s12917-025-04595-1. 40011950 10.1186/s12917-025-04595-1 PMC11866582

[36] Peng Z, Wang H, Liang W, Chen Y, Tang X, Chen H, Wu B. A capsule/lipopolysaccharide/MLST genotype D/L6/ST11 of Pasteurella multocida is likely to be strongly associated with swine respiratory disease in China. Arch Microbiol. 2018;200:107–18. 10.1007/s00203-017-1421-y. 28825122 10.1007/s00203-017-1421-y

[37] Krieger A-K, Öhlmann S, Mayer L, Weiße C, Rieckmann K, Baums CG. Porcine iucA + but rmpA- Klebsiella pneumoniae strains proliferate in blood of young piglets but are killed by IgM and complement dependent opsonophagocytosis when these piglets get older. Vet Microbiol. 2022;266:109361. 10.1016/j.vetmic.2022.109361. 35131553 10.1016/j.vetmic.2022.109361

[38] Rieckmann K, Seydel A, Szewczyk K, Klimke K, Rungelrath V, Baums CG. Streptococcus suis cps7: an emerging virulent sequence type (ST29) shows a distinct, IgM-determined pattern of bacterial survival in blood of piglets during the early adaptive immune response after weaning. Vet Res. 2018;49:48. 10.1186/s13567-018-0544-8. 29903042 10.1186/s13567-018-0544-8 PMC6003162

