# Supplementary figures and images for: Characterization of a Pasteurella multocida type A strain associated with a severe bronchopneumonia outbreak in gilts
Source: Porcine Health Manag. 2026 Apr 11;12:18. doi: 10.1186/s40813-026-00510-8 (PMC13072542; doi:10.1186/s40813-026-00510-8)

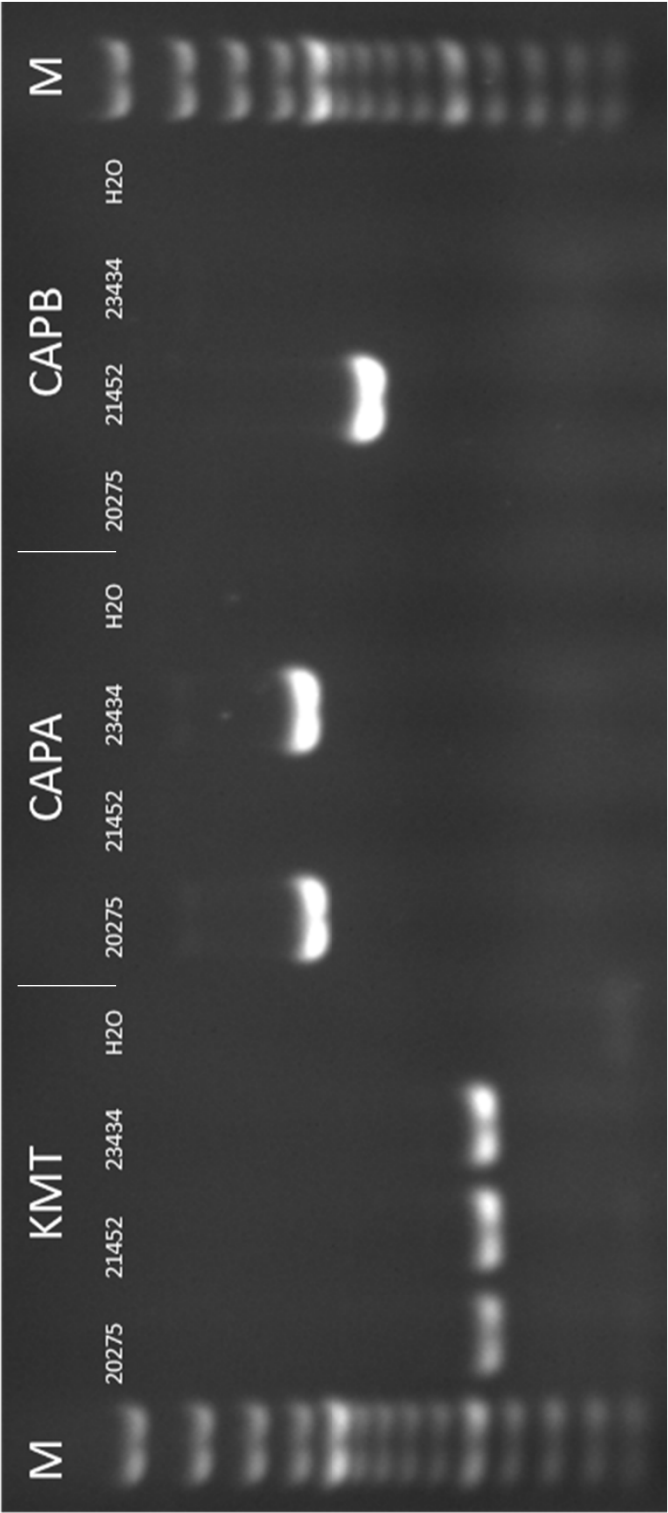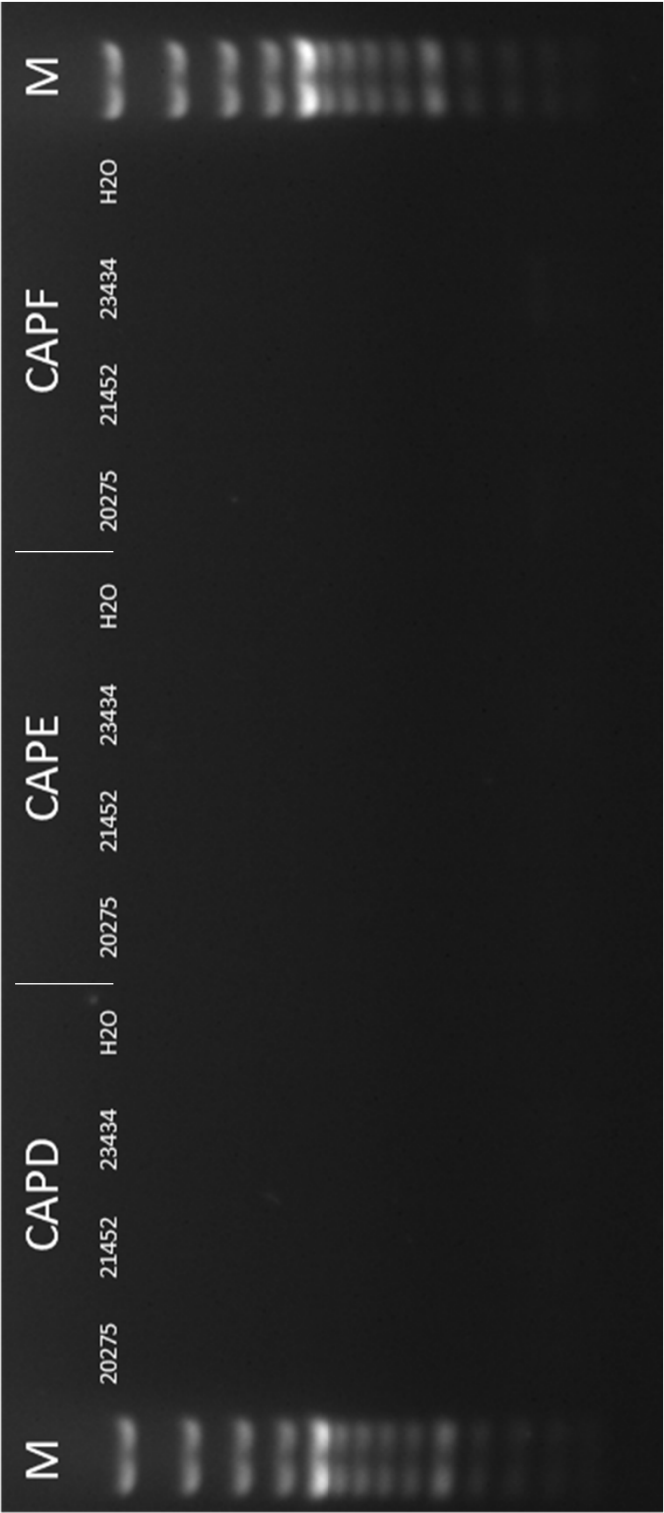

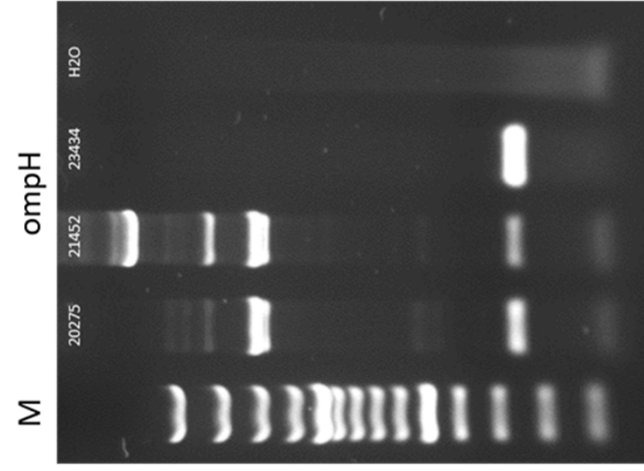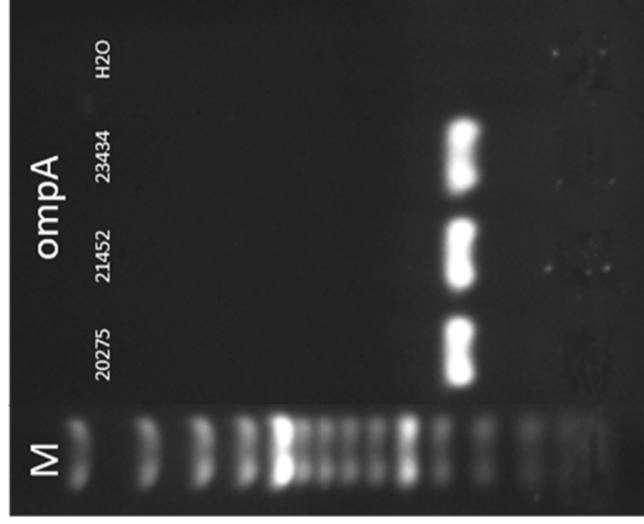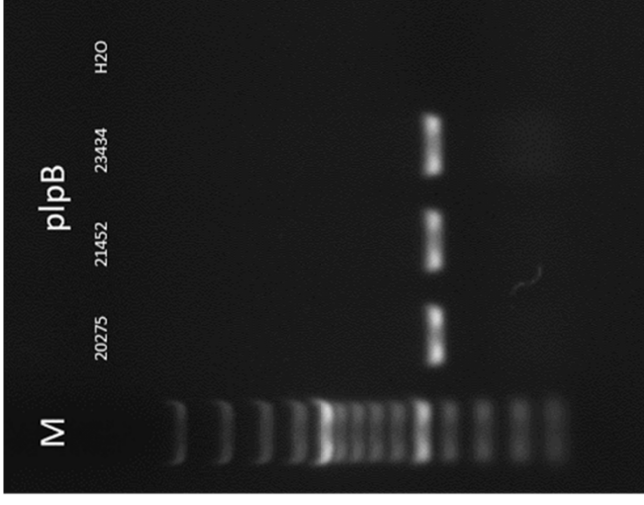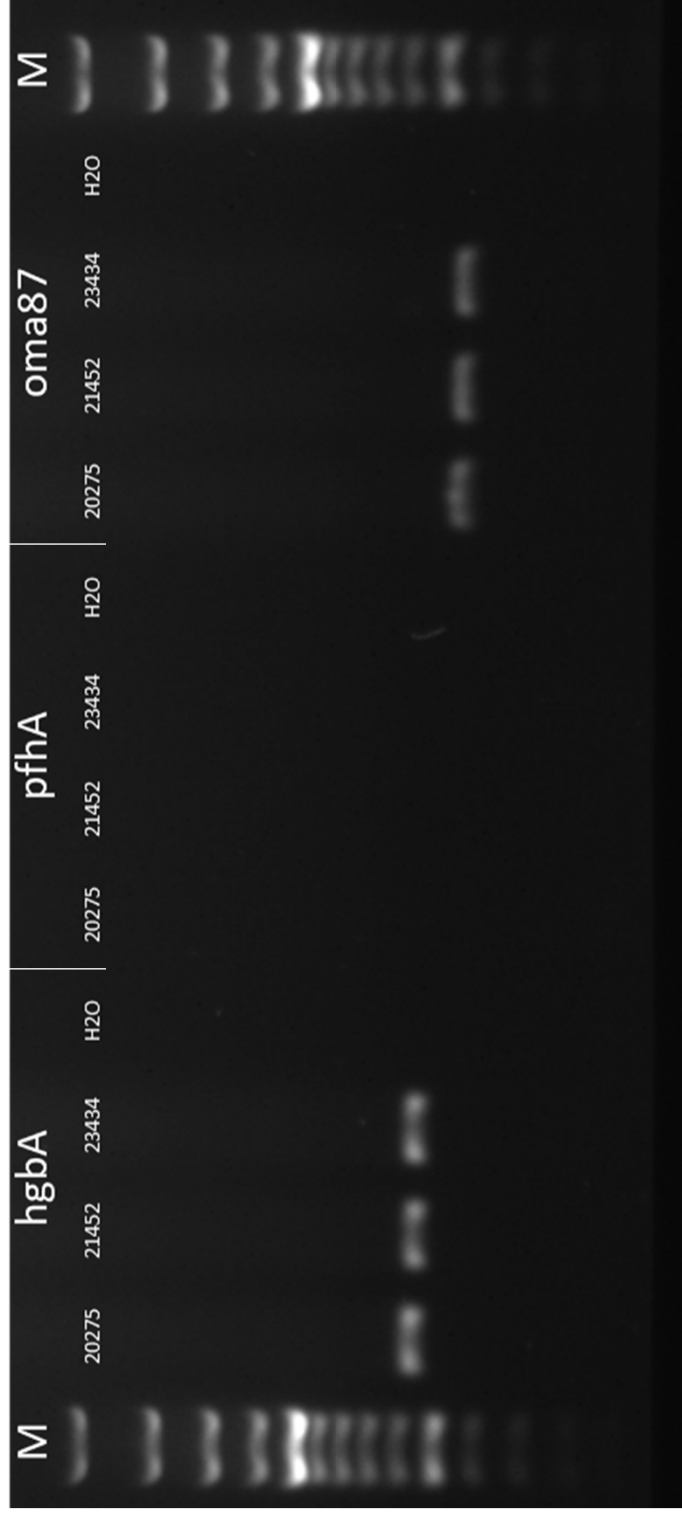

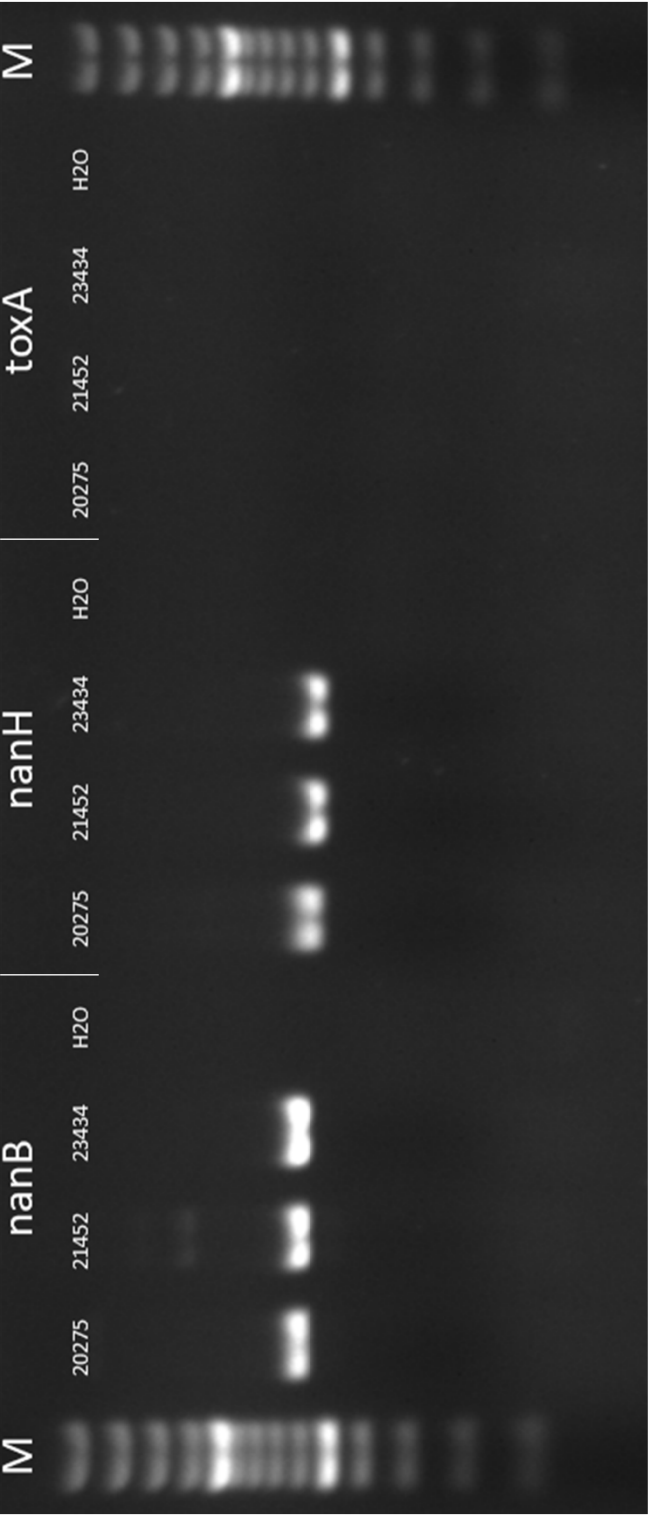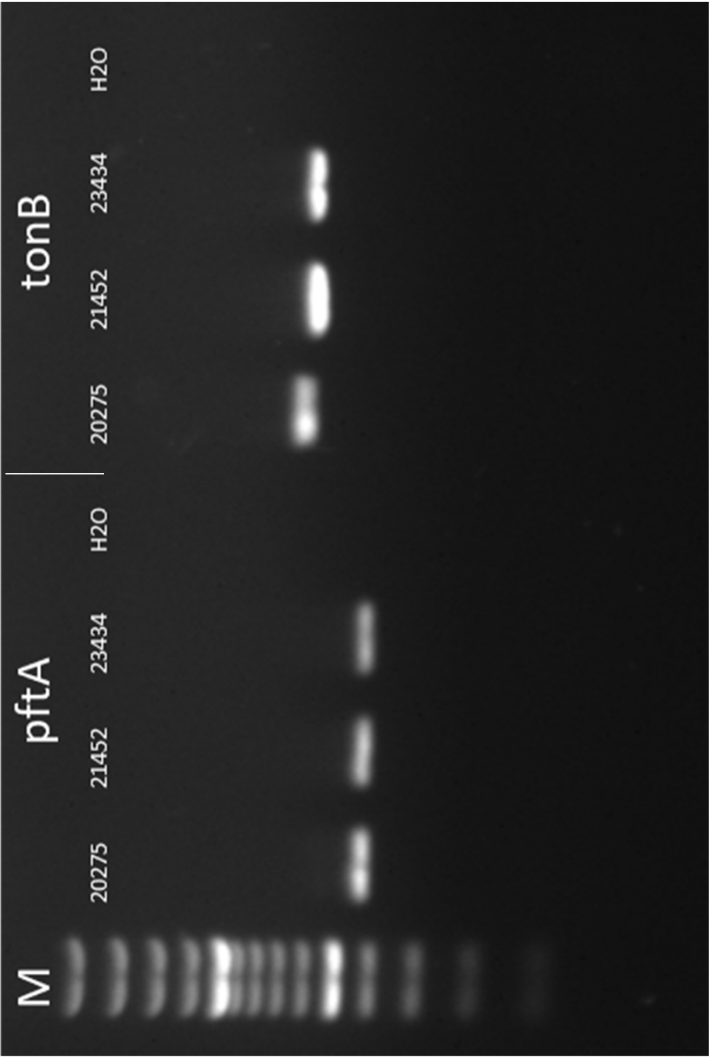

Supplement: Supplementary file 1 — Supplementary Material 1 [file 40813_2026_510_MOESM1_ESM.pdf]
